# Identification of key genes unique to the luminal a and basal-like breast cancer subtypes via bioinformatic analysis

**DOI:** 10.1186/s12957-020-02042-z

**Published:** 2020-10-16

**Authors:** Rong Jia, Zhongxian Li, Wei Liang, Yucheng Ji, Yujie Weng, Ying Liang, Pengfei Ning

**Affiliations:** College of Computer and Information, Inner Mongolia Medical University, Hohhot, 010110 Inner Mongolia Autonomous Region China

**Keywords:** Luminal a breast cancer, Basal-like breast cancer, Neoplasm genes, Bioinformatics

## Abstract

**Background:**

Breast cancer subtypes are statistically associated with prognosis. The search for markers of breast tumor heterogeneity and the development of precision medicine for patients are the current focuses of the field.

**Methods:**

We used a bioinformatic approach to identify key disease-causing genes unique to the luminal A and basal-like subtypes of breast cancer. First, we retrieved gene expression data for luminal A breast cancer, basal-like breast cancer, and normal breast tissue samples from The Cancer Genome Atlas database. The differentially expressed genes unique to the 2 breast cancer subtypes were identified and subjected to Gene Ontology and Kyoto Encyclopedia of Genes and Genomes pathway enrichment analyses. We constructed protein–protein interaction networks of the differentially expressed genes. Finally, we analyzed the key modules of the networks, which we combined with survival data to identify the unique cancer genes associated with each breast cancer subtype.

**Results:**

We identified 1114 differentially expressed genes in luminal A breast cancer and 1042 differentially expressed genes in basal-like breast cancer, of which the subtypes shared 500. We observed 614 and 542 differentially expressed genes unique to luminal A and basal-like breast cancer, respectively. Through enrichment analyses, protein–protein interaction network analysis, and module mining, we identified 8 key differentially expressed genes unique to each subtype. Analysis of the gene expression data in the context of the survival data revealed that high expression of *NMUR1* and *NCAM1* in luminal A breast cancer statistically correlated with poor prognosis, whereas the low expression levels of *CDC7*, *KIF18A*, *STIL*, and *CKS2* in basal-like breast cancer statistically correlated with poor prognosis.

**Conclusions:**

*NMUR1* and *NCAM1* are novel key disease-causing genes for luminal A breast cancer, and *STIL* is a novel key disease-causing gene for basal-like breast cancer. These genes are potential targets for clinical treatment.

## Background

Breast cancer comprises malignant tumors originating from mammary epithelial tissue. It is one of the most common cancers in women and the leading cause of cancer-related deaths in women globally [1]. In 2000, Perou et al. proposed the molecular classification of breast cancer to facilitate accurate diagnosis and treatment [2]. For the first time, breast cancer was classified into the following types: luminal-like, Her-2-positive, basal-like, and normal breast-like. In 2001, Sørlie et al. further classified luminal-like breast cancer into luminal A and luminal B subtypes and demonstrated that different subtypes of breast cancer were statistically associated with prognosis [3]: the luminal A subtype was associated with the best prognosis, followed by the luminal B subtype, whereas the Her-2-positive and basal-like subtypes were associated with the worst prognosis. Therefore, identifying the regulatory mechanisms of the breast cancer subtypes to develop targeted therapies is essential to achieve optimal results for individual patients.

Luminal A is the most common molecular subtype of breast cancer with a relatively good prognosis. Endocrine therapy is the preferred treatment option for luminal A breast cancer since the tumor is hormone receptor-positive [4]. However, several genetic factors determine the efficacy of endocrine therapy for luminal A breast cancer. *FOXA1* expression is associated with estrogen receptor (ER) positivity in luminal A breast cancer [5, 6]. Prognostic testing by Thangavelu et al. revealed that *CENPI* overexpression is a strong independent marker for ER-positive breast cancer that can be used to predict patient prognosis and survival [7]. They further demonstrated that *CENPI* is an E2F target gene. Karn and Emerson suggested that mutations in *GATA3* resulted in differential gene expression in ER-positive breast tumors, which affected prognosis [8]. In addition, Alfarsi et al. found that high *KIF18A* expression exhibited prognostic significance and could predict the adverse effects of endocrine therapy in patients with ER-positive breast cancer [9]. Thus, *KIF18A* testing of patients with ER-positive breast cancer prior to treatment could guide clinicians’ decision-making on whether the patients would benefit from endocrine therapy.

Basal-like breast cancer is associated with a poor prognosis. Due to the lack of effective therapeutic targets, the primary clinical treatment modality for basal-like breast cancer remains chemotherapy [10, 11]. Several groups have investigated the genetic profile of basal-like breast cancer to identify novel, specific targets to improve patient outcomes. Komatsu et al. identified cell cycle regulators, *ASPM*, and *CENPK* as potential disease-causing genes for basal-like breast cancer and utilized them as therapeutic targets in vitro [12]. Rodriguez-Acebes et al. demonstrated that the cell cycle gene *CDC7* may represent an effective, highly specific anticancer target in triple-negative breast cancer (TNBC) overexpressing Her-2 [13]. Song et al. used miRNA microarray to analyze 2 *BRCA1*-mutated TNBC cell lines [14]. They found that the addition of a PARP inhibitor to the carboplatin plus gemcitabine therapy regimen led to increased expression of miR-664b-5p and that *CCNE2* is a novel functional target of miR-664b-5p. Ye et al. showed that *CDCA7* upregulated *EZH2* transcripts and played a key role in TNBC progression, making *CDCA7* a potential prognostic factor and therapeutic target for TNBC [15].

Most of the aforementioned studies analyzed general breast cancer samples or a single subtype of breast cancer. Few comparative analyses of breast cancer subtypes have been reported. In the present study, we aimed to identify novel, more accurate targets for clinical treatment based on the comparative analysis of the genetic profiles and prognoses of patients with luminal A and basal-like breast cancer. We used bioinformatics to comprehensively analyze the gene expression data for each subtype and determine uniquely differentially expressed genes. Then, we utilized protein–protein interaction (PPI) network analysis to identify the key genes for each subtype. The key genes were used as specific markers for luminal A breast cancer and basal-like breast cancer for receiver operating characteristic (ROC) curve and survival analyses to identify prognosis-associated candidate genes for targeted breast cancer treatment.

## Methods

### Data

Gene expression data from 439 samples—comprising 255 luminal A breast cancer samples, 87 basal-like breast cancer samples, and 97 normal breast tissue samples—were downloaded from The Cancer Genome Atlas (TCGA) database [16].

### Differentially expressed gene analysis

We used R package limma [17] to compare the gene expression data from the luminal A breast cancer samples and normal breast tissue samples and between the basal-like breast cancer samples and normal breast tissue samples. The screening threshold was set to |log*FC*| > [mean(|log*FC*|) + 2*sd*(|log*FC*|)] with *P* value < 0.05, and the expression data were normalized based on Trimmed Mean of M value (TMM) in R package edgeR [18]. We used the R packages clusterProfiler [19] and org.H.eg.db to convert the IDs of the differentially expressed genes into gene names. Finally, the R package plot was used to create volcano plots of the differentially expressed genes.

To identify the common and unique differentially expressed genes in luminal A and basal-like breast cancer, we compared the differentially expressed genes identified in the 2 subtypes with R package dplyr [20]. We selected the common differentially expressed genes in the 2 subtypes with opposite modes of expression.

### Enrichment analyses of differentially expressed genes

To analyze the unique biological processes in the pathogenesis of luminal A and basal-like breast cancer, we performed enrichment analyses with R package clusterprofiler. Gene ontology (GO) enrichment analysis categorized genes as related to biological processes, cellular components, or molecular functions, and Kyoto Encyclopedia of Genes and Genomes (KEGG) [21] analysis categorized genes based on pathway enrichment. We generated bubble charts of the functions and pathways of the differentially expressed genes to facilitate the interpretation of the biological significance of the genes.

### Construction of PPI networks of differentially expressed genes

We used the online tool STRING [22] to obtain the PPI networks of the differentially expressed genes unique to luminal A and basal-like breast cancer. Isolated protein nodes with no interactions with other proteins were eliminated. We used Cytoscape software to visualize the PPI networks and the plugin MCODE to screen for the functional modules of the networks. The screening criterion was an MCODE score of ≥ 5. The cytoHubba plugin was used to identify key genes with the following settings: Hubba_nodes=8, Ranking Method=“DMNC.” We used the online tool DAVID [23] for KEGG pathway enrichment analysis of the key genes. A difference with a *P* value < 0.05 was considered significant.

### Analysis of prognostic value

We used the key genes as specific markers for luminal A and basal-like breast cancer in ROC curve analyses. The key genes were further analyzed for associations with survival using the online tool PROGgeneV2 [24]. The settings were SURVIVAL MEASURE=“MEDIAN” and SURVIVAL MEASURE=“DEATH.” We obtained survival curves for patients with luminal A and basal-like breast cancer subtypes who had different expression levels of the unique key genes. A difference with a *P* value < 0.05 was considered significant.

## Results

### Identification of differentially expressed genes

**Fig. 1:**
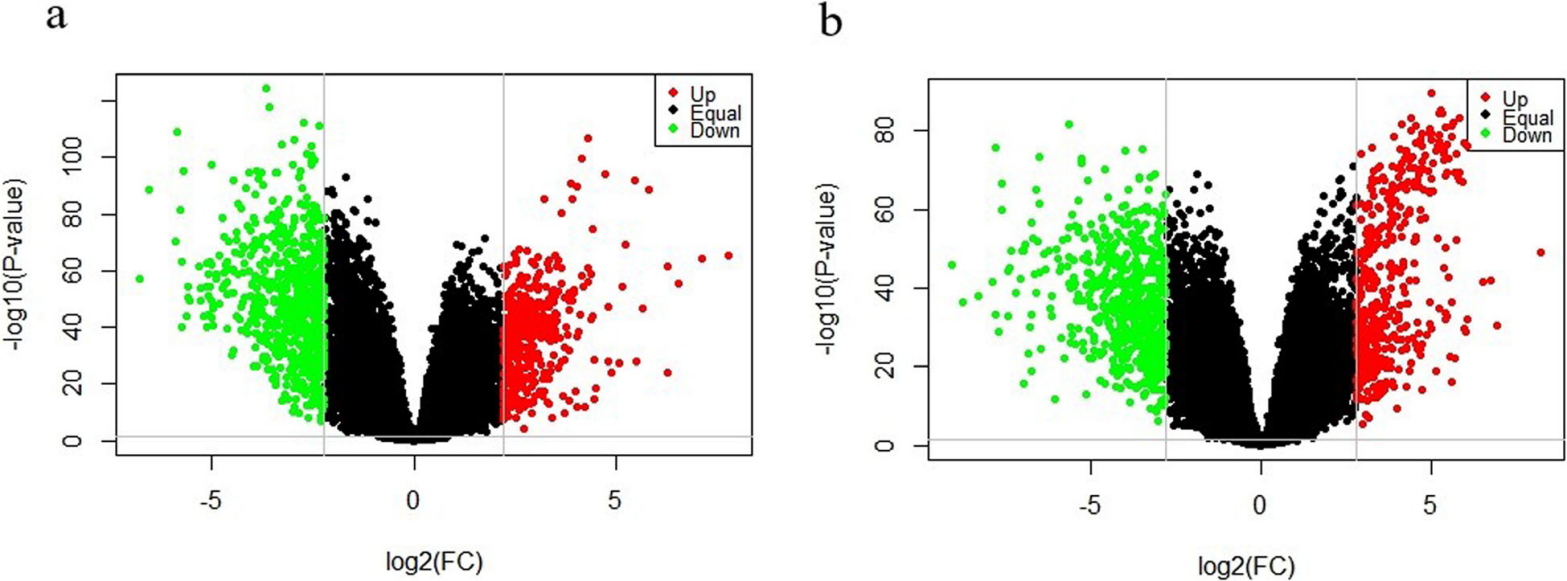
Differentially expressed genes in luminal A and basal-like breast cancer versus normal breast tissue. **a** Volcano plot of the differentially expressed genes identified by comparison of 255 luminal A breast cancer samples and 97 normal breast tissue samples. The 453 upregulated genes are shown in red (Up), and the 661 downregulated genes are shown in green (Down). **b** Volcano plot of the differentially expressed genes identified by comparison of 87 basal-like breast cancer samples and 97 normal breast tissue samples. The 435 upregulated genes are shown in red (Up), and the 607 downregulated genes are shown in green (Down). Genes that were not differentially expressed are shown in black (Equal)

We performed a differential analysis of 255 samples of luminal A breast cancer and 97 samples of normal breast tissue. Based on the criteria |log*FC*| > 2.199 and *P* value < 0.05, we identified 1114 differentially expressed genes, including 453 upregulated genes and 661 downregulated genes, from a total of 20,531 genes (Fig. 1). We also performed a differential analysis of 87 samples of basal-like breast cancer and 97 samples of normal breast tissue. Based on the criteria |log*FC*| > 2.799 and *P* value < 0.05, we selected 1042 differentially expressed genes, including 435 upregulated genes and 607 downregulated genes, from a total of 20,531 genes (Fig. 1).

**Fig. 2:**
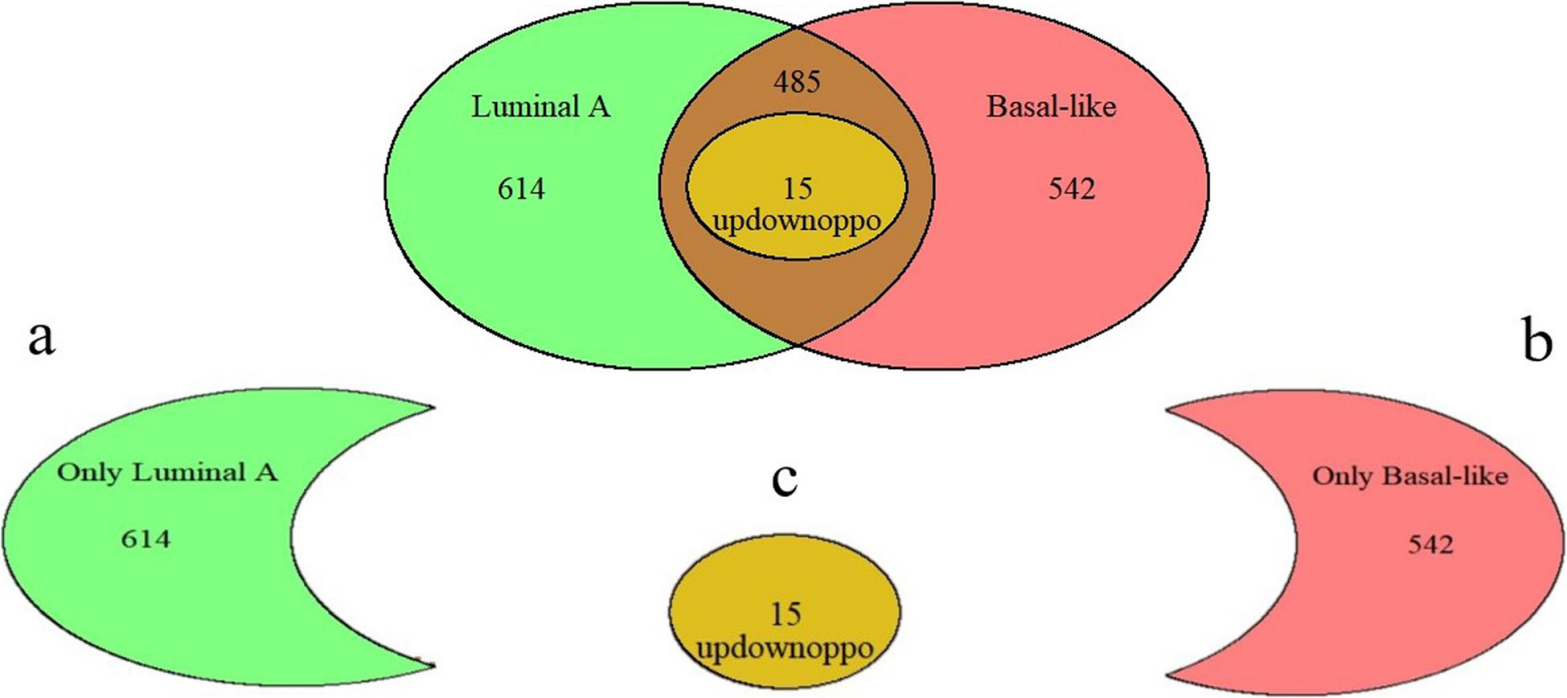
Expression patterns of the differentially expressed genes in luminal A and basal-like breast cancer. **a** Luminal A breast cancer had 614 unique differentially expressed genes. **b** Basal-like breast cancer had 542 unique differentially expressed genes. **c** The subtypes shared 15 common differentially expressed genes with opposite expression patterns (updownoppo)

We compared the differentially expressed genes in the 2 breast cancer subtypes and found that 614 differentially expressed genes were unique to the luminal A breast cancer samples and 542 were unique to basal-like breast cancer samples (Fig. 2). The subtypes shared 500 differentially expressed genes. We identified 15 differentially expressed genes with opposite expression patterns in the luminal A and basal-like breast cancer samples. The relationships of the differentially expressed genes are shown in Fig. 2.

### Function and pathway enrichment analyses

**Fig. 3:**
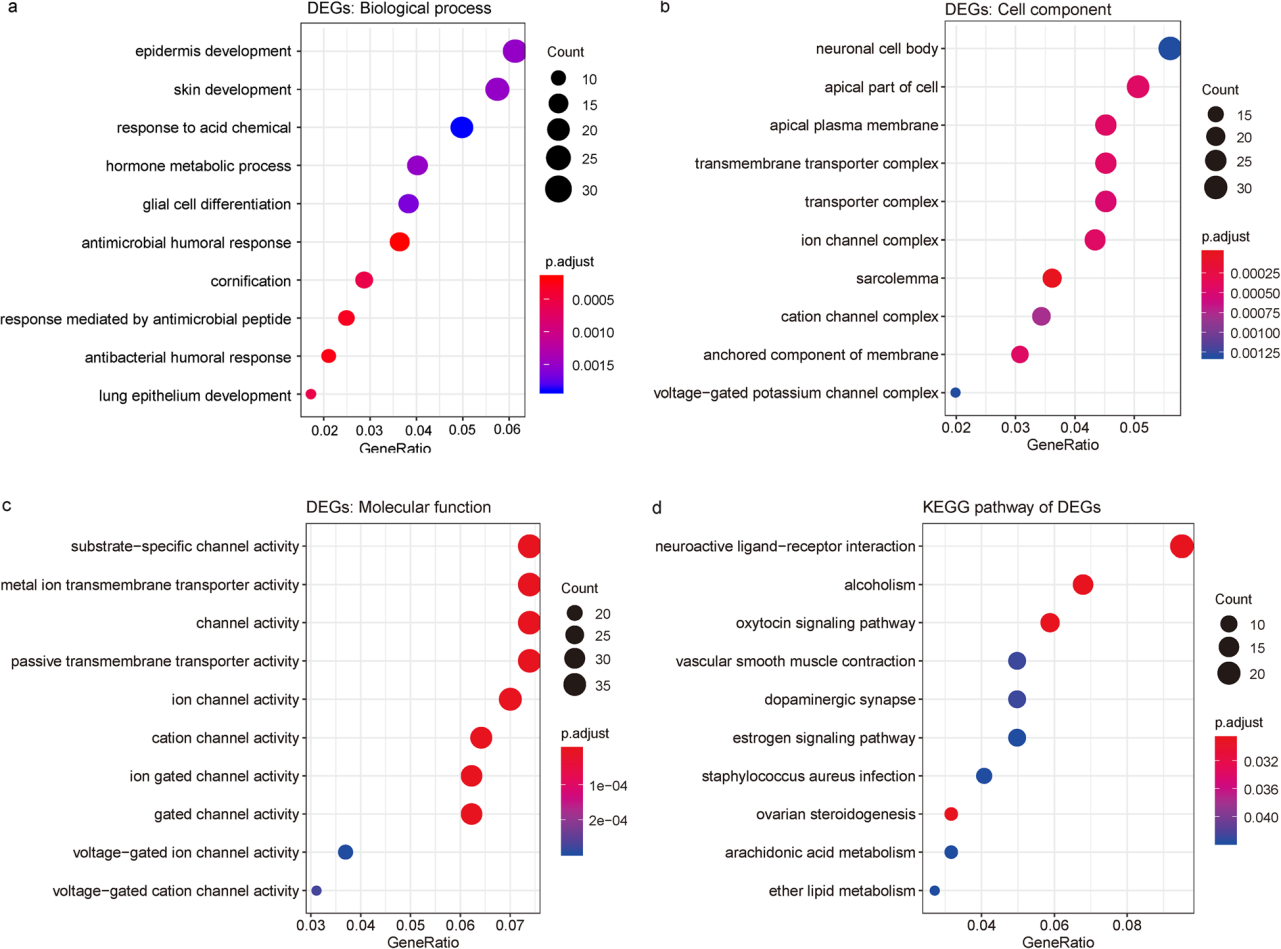
Enrichment analyses of the differentially expressed genes in luminal A breast cancer. **a** GO enrichment analysis of biological processes. **b** GO enrichment analysis of cellular components. **c** GO enrichment analysis of molecular functions. **d** KEGG pathway analysis. P.adjust is the adjusted value of *P* value

GO enrichment analysis (Fig. 3) revealed that the 614 differentially expressed genes unique to luminal A breast cancer were mainly involved in biological processes, including the antimicrobial humoral response, epidermis development, glial cell differentiation, and hormone metabolic process. The differentially expressed genes with a relationship to cellular components were significantly associated with multiple components, such as the sarcolemma, apical plasma membrane, ion channel complex, transmembrane transporter complex, and neuronal cell body. The significantly enriched molecular functions of the differentially expressed genes included cation channel activity, substrate-specific channel activity, metal ion transmembrane transporter activity, and passive transmembrane transporter activity. In addition, the significantly enriched KEGG pathways comprised the oxytocin signaling pathway, neuroactive ligand–receptor interaction, ovarian steroidogenesis, vascular smooth muscle contraction, dopaminergic synapses, *Staphylococcus aureus* infection, and estrogen signaling pathway (Fig. 3).

**Fig. 4:**
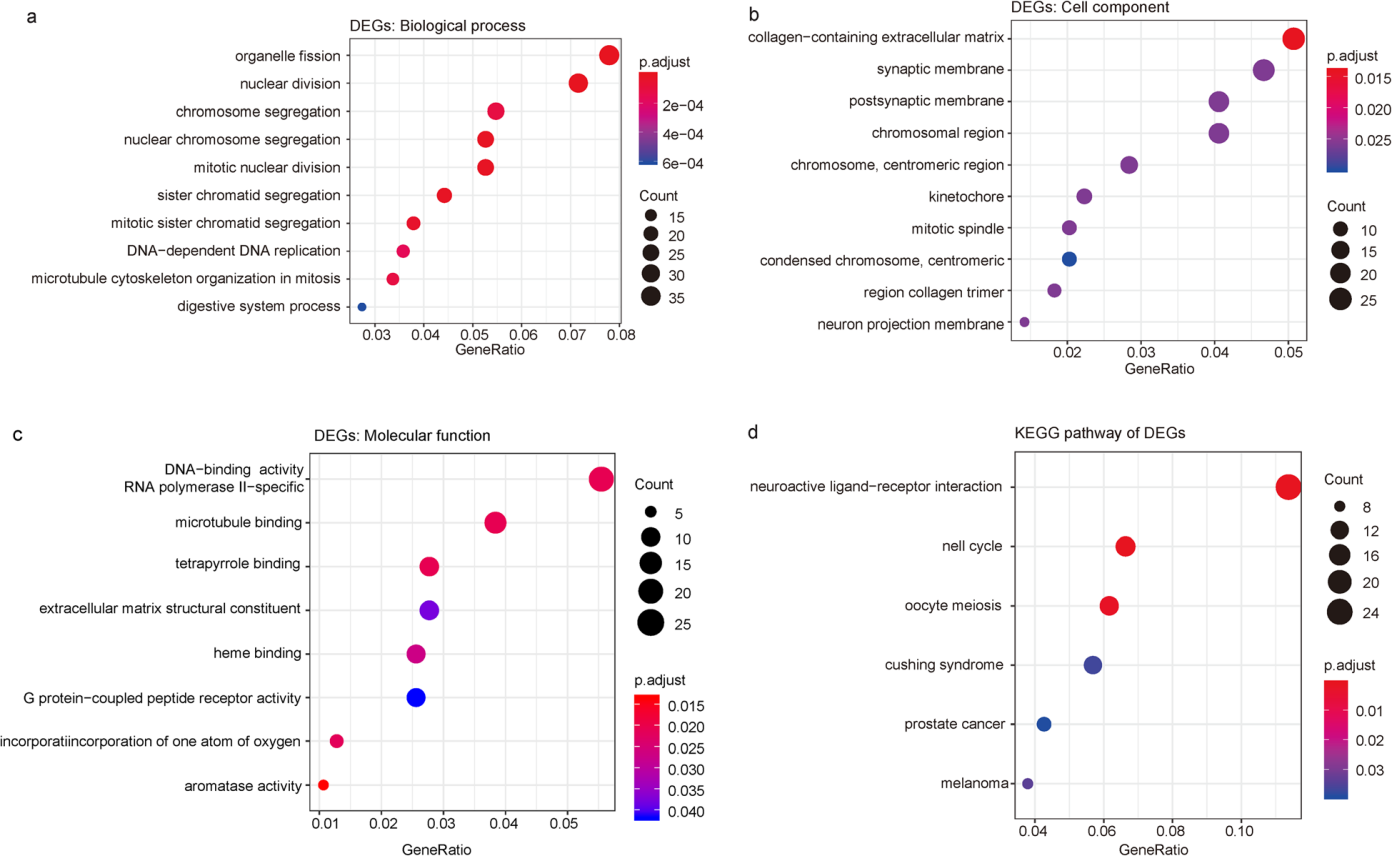
Enrichment analyses of the differentially expressed genes in basal-like breast cancer. **a** GO enrichment analysis of biological processes. **b** GO enrichment analysis of cellular components. **c** GO enrichment analysis of molecular functions. **d** KEGG pathway analysis. P.adjust is the adjusted value of *P* value

GO enrichment analysis (Fig. 4) revealed that the 542 differentially expressed genes unique to basal-like breast cancer were mainly involved in biological processes, including organelle fission, nuclear division, nuclear chromosome segregation, sister chromatid segregation, mitotic nuclear division, chromosomal segregation, and DNA-dependent DNA replication. The differentially expressed genes were significantly associated with multiple cell components, such as collagen-containing extracellular matrix, postsynaptic membrane, collagen trimer, and chromosomal and centromeric regions. The significantly enriched molecular functions of the differentially expressed genes included aromatase activity, RNA polymerase II-specific DNA-binding transcription activation activity, oxidoreductase activity, and G protein-coupled peptide receptor activity. In addition, the significantly enriched KEGG pathways were cell cycle, neuroactive ligand–receptor interaction, oocyte meiosis, melanoma, and Cushing syndrome. The detailed results of the analyses are shown in Fig. 4.

### PPI network construction

**Fig. 5:**
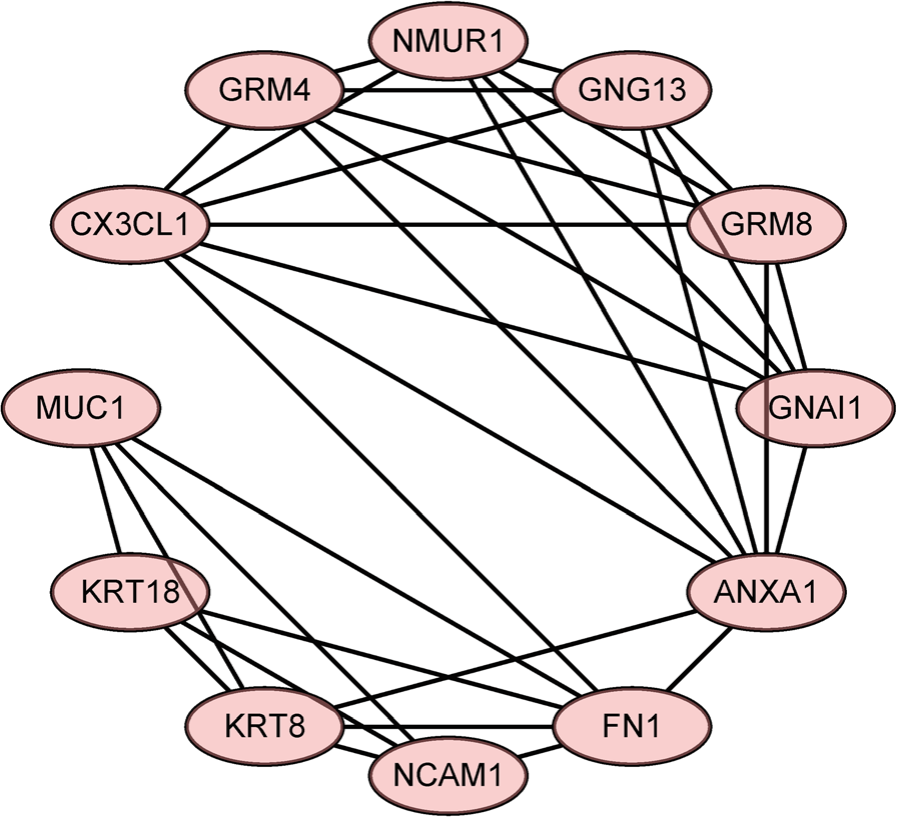
Luminal A breast cancer module

Next, we sought to further understand the functional modules in the PPI networks of the differentially expressed genes unique to luminal A and basal-like breast cancer to identify the key genes for each disease. The MCODE Cytoscape plugin was used to construct the functional modules in the PPI network of the differentially expressed genes unique to luminal A breast cancer. Functional modules with scores > 5 were selected. The module in Fig. 5 has a score of 6.182 and contains 12 nodes and 24 edges.

**Fig. 6:**
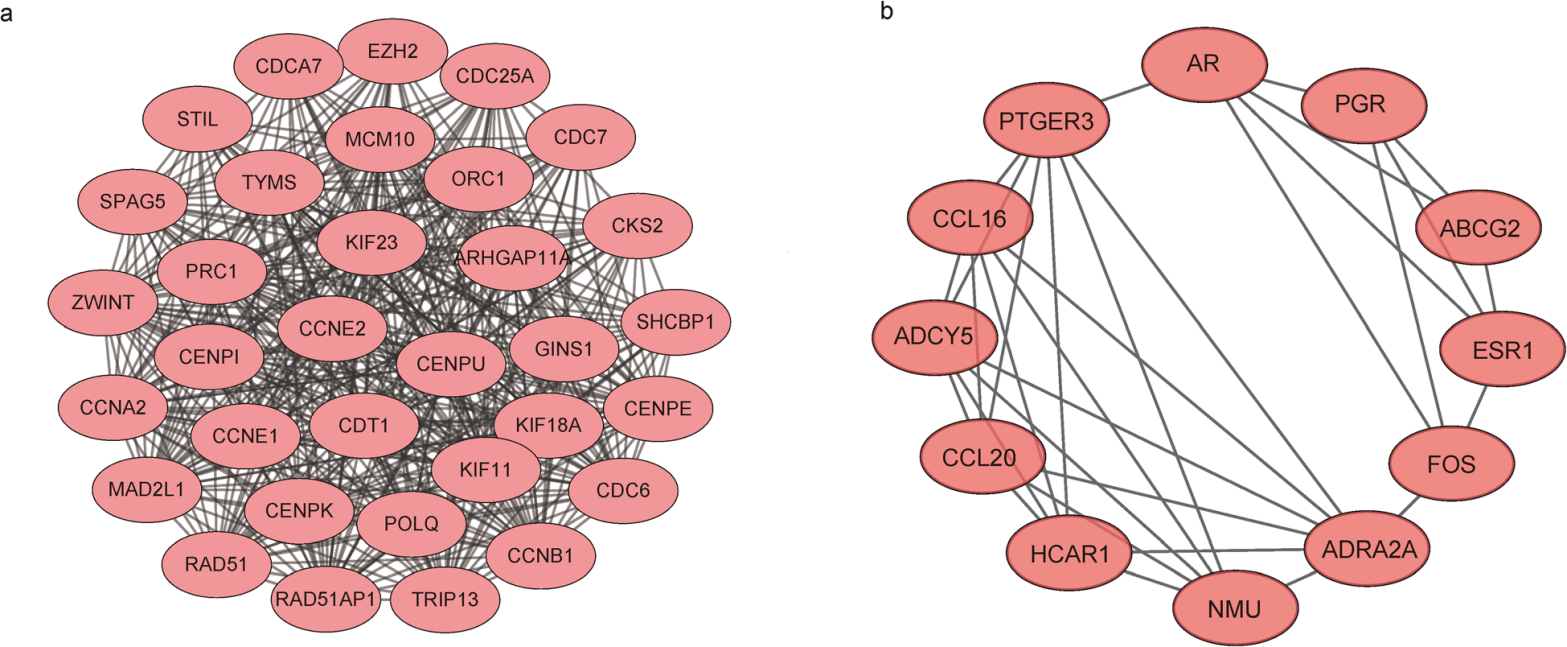
Modules in the PPI network of differentially expressed genes in the basal-like breast cancer subtype. **a** PPI network module 1. **b** PPI network module 2

We similarly constructed functional modules in the PPI network of the differentially expressed genes unique to basal-like breast cancer. Module 1 has a score of 25.812 and contains 33 nodes and 413 edges; module 2 has a score of 5.818 and contains 12 nodes and 32 edges (Fig. 6).

**Fig. 7:**
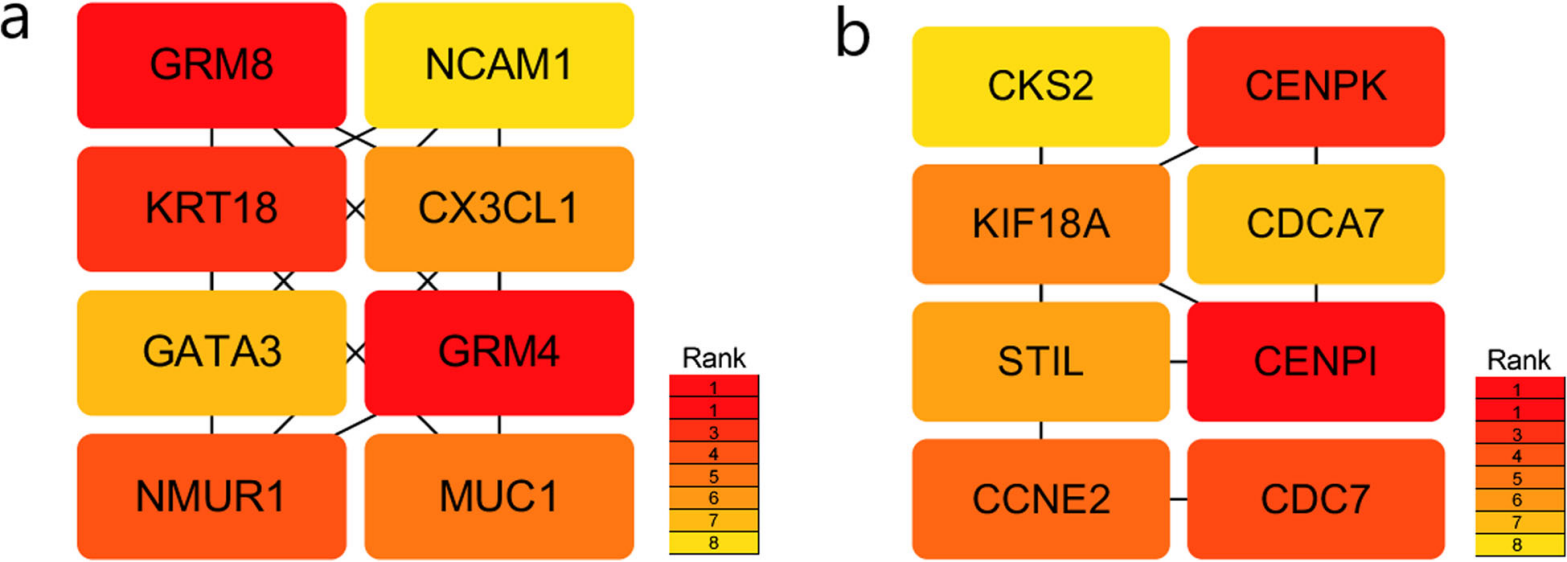
Key subtype-specific genes. **a** Key genes for luminal A breast cancer. **b** Key genes for basal-like breast cancer

We further analyzed the above modules to find the hub genes. We used the cytoHubba Cytoscape plugin (settings: Hubba_nodes=8, Ranking Method=“DMNC”) to screen for 8 key genes respectively among the luminal A breast cancer module and the basal-like breast cancer module 1 (Fig. 7). The key genes identified for luminal A breast cancer were *GRM4*, *GRM8*, *KRT18*, *NMUR1*, *MUC1*, *CX3CL1*, *GATA3*, and *NCAM1*. The neuroactive ligand–receptor interaction pathway was enriched for *GRM4*, *NMUR1*, and *GRM8* (*P* < 0.05). The metabotropic glutamate receptors were a family of G protein-coupled receptors that had been divided into 3 groups on the basis of sequence homology, putative signal transduction mechanisms, and pharmacologic properties. *GRM8* and *GRM4* belong to group III that receptors were linked to the inhibition of the cyclic AMP cascade but differed in their agonist selectivities [25]. *KRT18* encoded the type I intermediate filament chain keratin 18. Keratin 18, together with its filament partner keratin 8, were perhaps the most commonly found members of the intermediate filament gene family. They were expressed in single layer epithelial tissues of the body [26]. Neuromedin U Receptor 1 (*NMUR1*) was a protein coding gene. Among its related pathways were RET signaling and signaling by *GPCR*. Gene Ontology (GO) annotations related to this gene included G protein-coupled receptor activity and neuromedin U receptor activity. An important paralog of this gene was *NMUR2* [27]. The key genes identified for basal-like breast cancer were *CENPI*, *CENPK*, *CDC7*, *CCNE2*, *KIF18A*, *STIL*, *CDCA7*, and *CKS2*. The small cell lung cancer pathway was enriched for *CCNE2* and *CKS2*, and the cell cycle pathway was enriched for *CDC7* and *CCNE2* (*P* < 0.05). *CENPI* encoded a centromere protein that was a component of the CENPA-NAC (nucleosome-associated) complex. This protein regulated the recruitment of kinetochore-associated proteins that were required to generate the spindle checkpoint signal. The product of this gene was involved in the response of gonadal tissues to follicle-stimulating hormone [28]. *CENPK* was a subunit of a CENPH-CENPI-associated centromeric complex that targeted *CENPA* to centromeres and was required for proper kinetochore function and mitotic progression [29]. The protein encoded by *CCNE2* belongs to the highly conserved cyclin family, whose members were characterized by a dramatic periodicity in protein abundance through the cell cycle. Cyclins function as regulators of CDK kinases [30]. *CDC7* encoded a cell division cycle protein with kinase activity that was critical for the G1/S transition. Overexpression of this gene product might be associated with neoplastic transformation for some tumors [31].

### Analysis of prognostic value

**Fig. 8:**
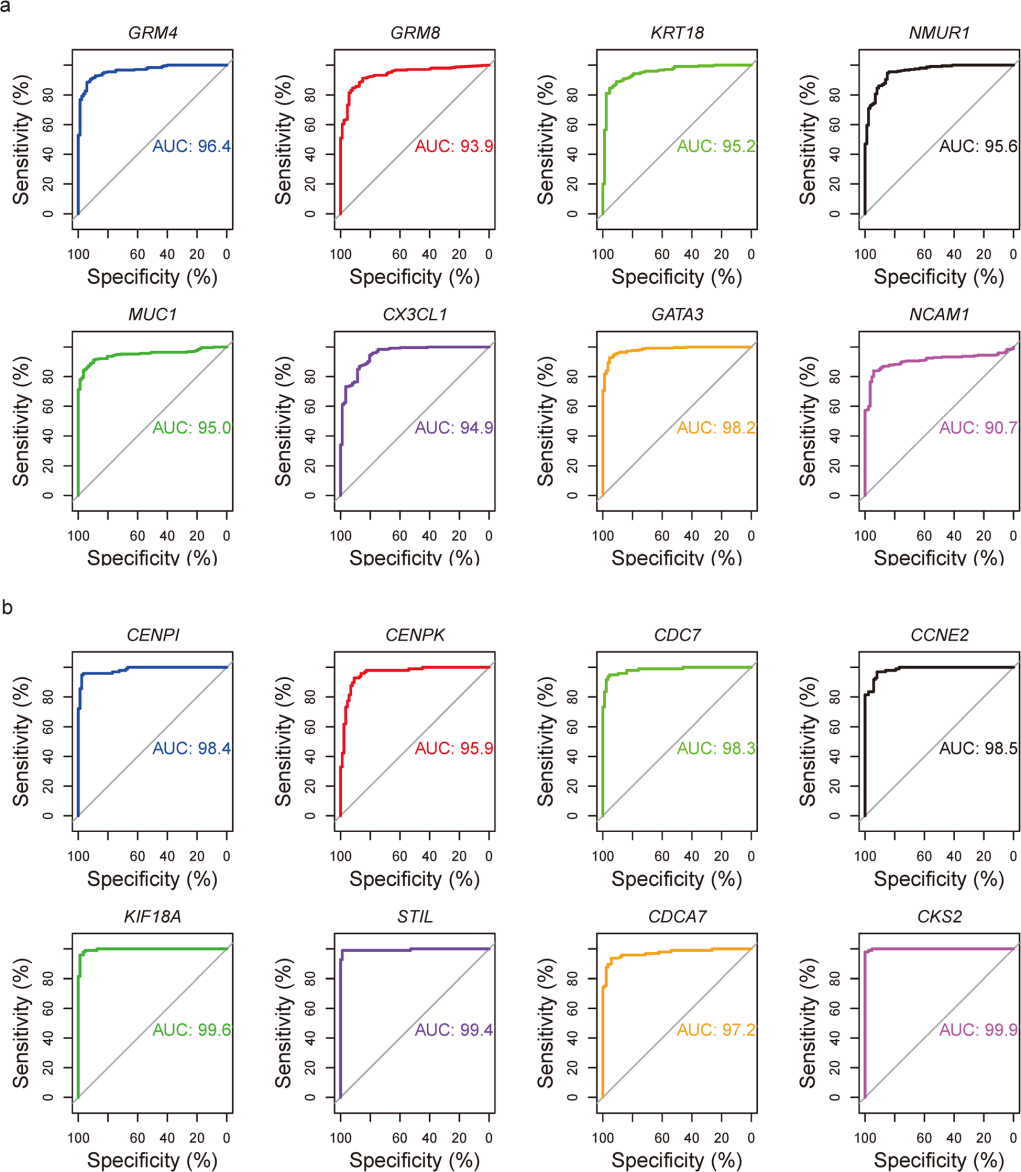
ROC curves of the key genes. **a** ROC curves of the key genes, including *GRM4*, *GRM8*, *KRT18*, *NMUR1*, *MUC1*, *CX3CL1*, *GATA3*, and *NCAM1*, for luminal A breast cancer. **b** ROC curves of the key genes, including *CENPI*, *CENPK*, *CDC7*, *CCNE2*, *KIF18A*, *STIL*, *CDCA7*, and *CKS2*, for basal-like breast cancer

We created ROC curves for the 2 sets of key genes. ROC curve analysis showed that these genes exhibited good prognostic value for their associated cancer subtypes. The areas under the ROC curves were greater than 90% for all genes, as shown in Fig. 8.

**Fig. 9:**
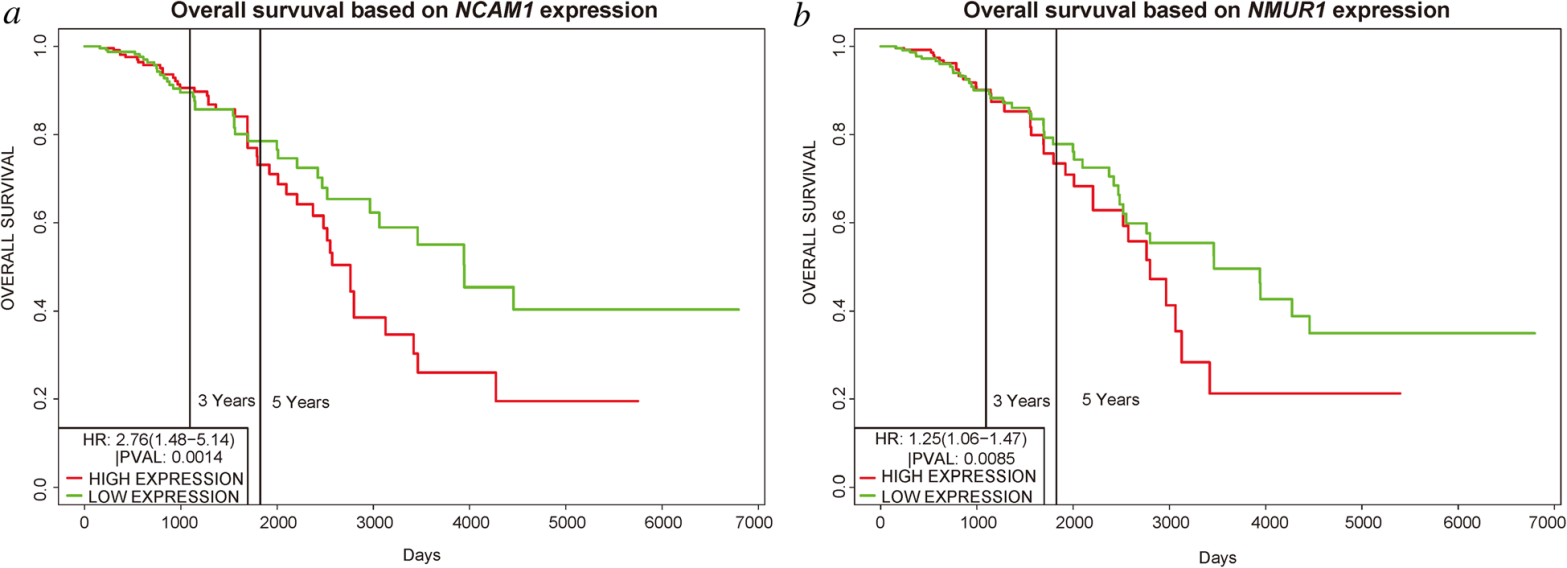
Survival of patients with luminal A breast cancer by expression of key genes. **a** *NMUR1*. **b** *NCAM1*. Survival and gene expression data were retrieved from TCGA. The cohort was divided at the median gene expression (*P* < 0.05)

The prognostic values of the selected key genes unique to luminal A breast cancer were analyzed using the PROGgeneV2 online tool. We retrieved the survival curves of the patients from the TCGA database with the corresponding breast cancer subtype and analyzed survival by the expression levels of the key genes (Fig. 9). Of the key genes unique to the luminal A breast cancer subtype, the expression levels of only *NMUR1* and *NCAM1* were associated with patient survival time (*P* < 0.05). Survival analysis showed that higher expression levels of the prognosis-related key genes were associated with shorter survival time of luminal A breast cancer patients.

**Fig. 10:**
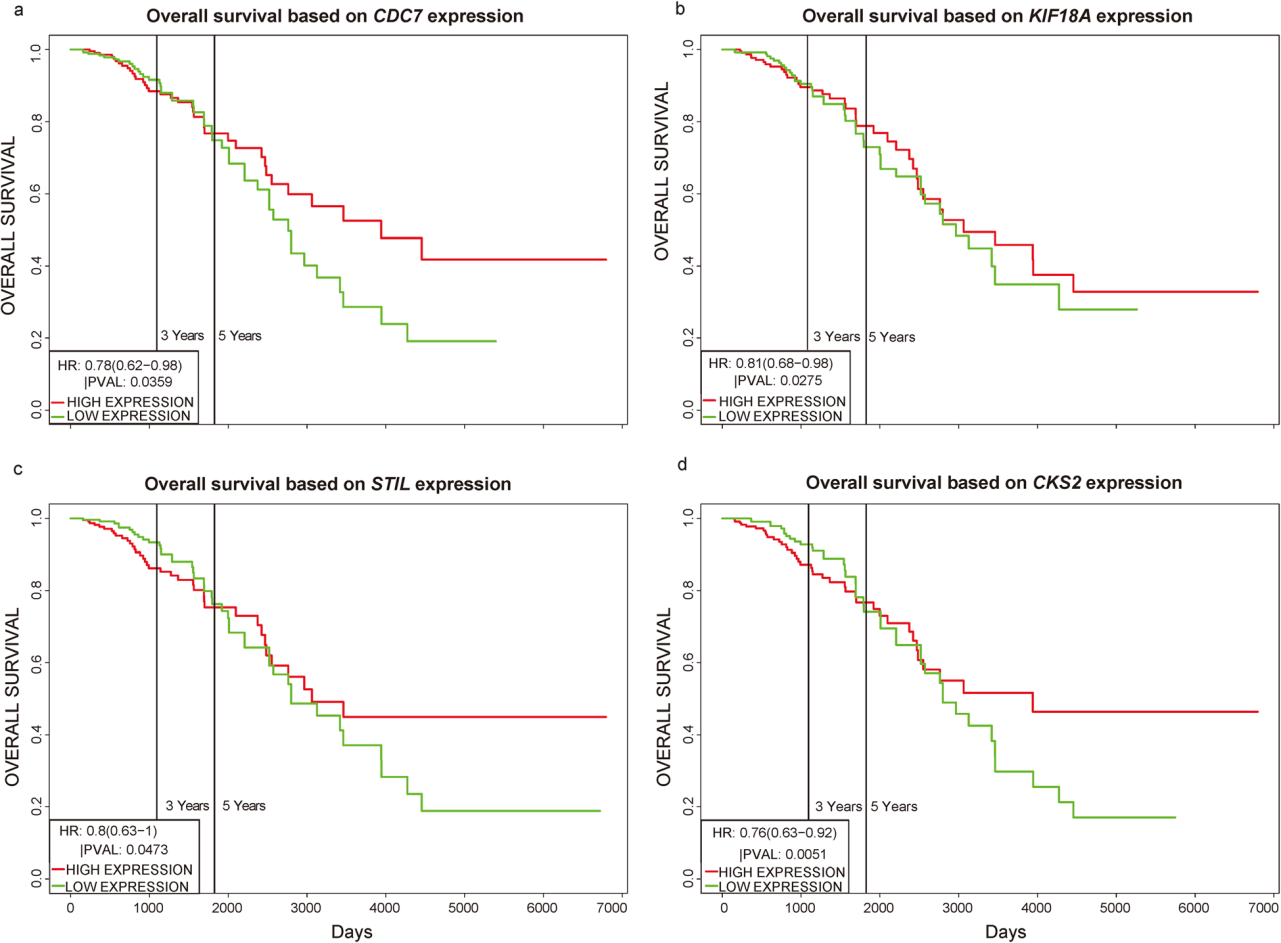
Survival of patients with basal-like breast cancer by expression of key genes. **a** *CDC7*. **b** *KIF18A*. **c** *STIL*. **d** CKS2. Survival and gene expression data were retrieved from TCGA. The cohort was divided at the median gene expression (*P* < 0.05)

Next, we used the same methodology to analyze the prognostic values of the key genes unique to basal-like breast cancer (Fig. 10). Of the key genes unique to basal-like breast cancer, the expression levels of only *CDC7*, *KIF18A*, *STIL*, and *CKS2* were associated with patient survival time (*P* < 0.05). Lower than median expression levels of the prognosis-related key genes were associated with better prognosis

.

## Discussion

Breast cancer is the most common malignancy, which with high heterogeneity in terms of the underlying molecular alterations. Different subtypes exhibit distinct biological behavior, prognosis and treatment. Although the treatment of breast cancer has improved, the prognosis of patients is still poor. Herein, it is urgently needed to identify precise therapeutic targets for different subtypes of breast cancer [3].

In our studies, we identify new molecular targets of luminal A breast cancer and basal-like breast cancer using bioinformatic analysis. We identified *NMUR1* and *NCAM1* as novel key genes associated with the development, progression, and prognosis of luminal A breast cancer, and *STIL* as a novel key gene associated with the prognosis of basal-like breast cancer.

*NMUR1*, which is associated with the poor prognosis of luminal A breast cancer patients, encodes the neuromedin U receptor 1. *NMUR1* is broadly expressed in human tissues, with the highest expression in the adipose tissue, intestine, spleen, and lymphocytes. It likely possesses physiological effects that remain to be elucidated [32]. *NCAM1* encodes neural cell adhesion molecule 1. It is broadly used as a marker of minimal residual disease and is expressed in most acute myeloid leukemia molecular subgroups with high levels of heterogeneity. Sasca et al. used complementary genetic strategies to demonstrate the important role of *NCAM1* in the regulation of cell survival and stress resistance [33]. The roles of *NMUR1* and *NCAM1* in breast cancer have not been reported. We found that luminal A breast cancer patients with high expression of *NMUR1* and *NCAM1* had statistically worse overall survival than patients with below-median levels of expression. Thus, we predict that inhibition of *NMUR1* and *NCAM1* could represent a novel strategy to improve the treatment of luminal A breast cancer. STIL, also known as STIL centriolar assembly protein, encodes a cytoplasmic protein involved in the regulation of mitotic spindle checkpoints [34]. Ouyang et al. found that STIL expression was upregulated in various human tumor tissues and that higher expression of STIL was associated with shorter survival [35]. Our survival analysis showed that low expression of STIL statistically affects the overall survival of patients with basal-like breast cancer. Thus, we predict that promoting STIL expression could represent a novel modality to improve the treatment of basal-like breast cancer.

There are also several limitations in our study. We preliminarily explored the molecular mechanisms of these genes. The accuracy of new molecular targets needed for further experiments confirmed. In the future, we will investigate the expression of these oppositely regulated genes with the aim of developing specific therapies for the breast cancer subtypes.

## Conclusions

In summary, we identified *NMUR1* and *NCAM1* as novel key genes associated with the development, progression, and prognosis of luminal A breast cancer, and *STIL* as a new target with the prognosis of basal-like breast cancer.

## Data Availability

The data analyzed during the current study are available from the corresponding author on reasonable request.

## Abbreviations

ER: Estrogen receptor
TNBC: Triple-negative breast cancer
TMM: Trimmed Mean of M value
PPI: Protein–protein interaction
ROC: Receiver operating characteristic
TCGA: The Cancer Genome Atlas
GO: Gene Ontology
KEGG: Kyoto Encyclopedia of Genes and Genomes
